# Time patterns of recurrence and second primary tumors in a large cohort of patients treated for oral cavity cancer

**DOI:** 10.1002/cam4.2124

**Published:** 2019-08-10

**Authors:** Maria T. Brands, Elisabeth A. J. Smeekens, Robert P. Takes, Johannes H. A. M. Kaanders, Andre L. M. Verbeek, Matthias A. W. Merkx, Sandra M. E. Geurts

**Affiliations:** ^1^ Department of Oral and Maxillofacial Surgery Radboud University Medical Centre Nijmegen The Netherlands; ^2^ Department of Ear Nose and Throat Surgery Radboud University Medical Centre Nijmegen The Netherlands; ^3^ Department of Radiation Oncology Radboud University Medical Centre Nijmegen The Netherlands; ^4^ Department for Health Evidence Radboud University Medical Center Nijmegen The Netherlands; ^5^ Present address: Department of Oral and Maxillofacial Surgery Queen Elizabeth Hospital Queen Elizabeth University Hospital Glasgow United Kingdom; ^6^ Present address: Department of Surgery ETZ Hospital Tilburg The Netherlands; ^7^ Present address: Department of Medical Oncology GROW‐School for Oncology and Developmental Biology Maastricht University Medical Center Maastricht The Netherlands

**Keywords:** head and neck cancer, oral cancer, postoperative surveillance, recurrence, routine follow‐up, second primary tumor

## Abstract

**Introduction:**

Routine follow‐up after curative treatment of patients with oral squamous cell carcinoma (OSCC) is common practice considering the high risk of second primaries and recurrences (ie second events). Current guidelines advocate a follow‐up period of at least 5 years. The recommendations are not evidence‐based and benefits are unclear. This is even more so for follow‐up after a second event. To facilitate the development of an evidence‐ and personalized follow‐up program for OSCC, we investigated the course of time until the second and subsequent events and studied the risk factors related to these events.

**Materials and methods:**

We retrospectively studied 594 OSCC patients treated with curative intent at the Head and Neck Cancer Unit of the Radboud University Medical Centre from 2000 to 2012. Risk of recurrence was calculated addressing death from intercurrent diseases as competing event.

**Results:**

The 1‐, 5‐ and 10‐year cumulative risks of a second event were 17% (95% CI:14%;20%), 30% (95% CI:26%;33%), and 37% (95% CI:32%;41%). Almost all locoregional recurrences occurred in the first 2 years after treatment. The incidence of second primary tumors was relatively stable over the years. The time pattern of presentation of third events was similar.

**Discussion:**

Our findings support a follow‐up time of 2 years after curative treatment for OSCC. Based on the risk of recurrence there is no indication for a different follow‐up protocol after first and second events. After 2 years, follow‐up should be tailored to the individual needs of patients for supportive care, and monitoring of late side‐effects of treatment.

## Graphical abstract

There is no rationale for a follow‐up of 5 years after treatment for oral cancer, as the great majority of recurrences occurs in the first 2 years and because the risk of second primary tumors is constant. Patients should receive a core follow‐up of 2 years which can be extended based on an individual patients needs.

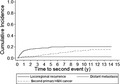

## 1 INTRODUCTION

Oral squamous cell carcinoma (OSCC) continues to be an important burden on health care, with an increasing incidence and only moderately improving survival.^1^, ^2^, ^3^, ^4^‐45% of OSCC patients will develop local‐ or regional recurrence (LRR), a second primary tumor (SPT) or distant metastasis (DM) (further called second events) after primary curative‐intent treatment.^4^, ^5^ Current guideline‐recommendations advocate follow‐up after curative treatment for all patients of at least 5 years.^6^ The main reason for follow‐up is the early detection of second events; other goals are functional rehabilitation and psychosocial support.

Follow‐up guidelines are not evidence‐ but consensus‐based. Empirical studies on follow‐up after treatment for OSCC are scarce and usually combine the data of all HNC's, which have a different etiology, treatment, prognosis, and timing of second events.^7^, ^8^, ^9^ The available studies on OSCC are small and do not address the question whether specific patient groups are in need of more or less intensive follow‐up.^10^, ^11^, ^12^, ^13^

The current “one‐size‐fits‐all” follow‐up programs can be criticized on several points.^14^, ^15^ Firstly, it is questionable if such a program is beneficial to all patients as some may be at higher risk of a second event than others.^16^, ^17^ The time frame of 5 years is debatable as most tumors seem to recur in the first few years.^7^, ^12^ Furthermore, it has never been investigated whether patients should receive a different follow‐up schedule after curative‐intent treatment of a second event.

It is of utmost importance to optimize and personalize OSCC follow‐up to avoid unnecessary testing and anxiety in patients, optimize the use of health care resources and minimize the time clinicians spend on ineffective follow‐up consultations. Therefore, this study investigates the time patterns, risks, and treatment intent of second and subsequent events after curative‐intent treatment of OSCC.

## 2 MATERIALS AND METHODS

### 2.1 Patients

Between 2000 and 2012, 756 patients were diagnosed with primary OSCC (ICD O codes C.00‐06 excluding C.01, C.05.1 en C.05.2) and treated at the Head and Neck Cancer Unit of the Radboud University Medical Centre. Of these patients, 57 were excluded from analysis for the following reasons: not a first primary OSCC (*n* = 23), a previous or synchronous tumor in other subsites of the head and neck area (*n* = 32) and other reasons (*n* = 2). Of the remaining 699 patients, 594 (85%) were treated with curative intent and eligible for analysis.

Patients were staged according to the seventh UICC TNM classification. Treatment intent and therapy choices were based on the Dutch national guideline. Decisions concerning therapy and treatment intent were taken after discussion in a multidisciplinary team meeting.^15^

Patients received follow‐up examinations every 2 and 3 months during the first and second year posttreatment, respectively, every 4 months in the third year and every 6 months during the fourth and fifth year. Survival was updated in November 2014 using the municipal registration of deaths.

The difference between a LRR and a SPT was based on p53 mutation analysis. If unavailable, the modified Warren and Gates criteria as described by Re et al were used.^18^, ^19^

### 2.2 Statistical analysis

Overall survival (OS) from the date of last primary treatment was calculated with the Kaplan‐Meier method. Median follow‐up time was determined by the inverse Kaplan‐Meier method (censored data as events).

Risk of recurrence was calculated using competing risk methods with death from intercurrent disease as competing event.^20^ Conditional risk of recurrence per follow‐up year was defined as the probability of experiencing a recurrence in that year (*y*), given that the patient had been recurrence‐free up to the previous follow‐up year (*x*). Annual conditional risk of event is calculated by dividing the cumulative risk of event‐free survival at “*x* + y” years after primary treatment by the cumulative risk at “*x*” years after treatment.^21^ Risk estimates are given with 95% confidence intervals (CI).

Independent prognostic factors were selected through forward stepwise regression with *P* < 0.10 as a cutoff. The Fine and Gray modified Cox proportional hazards model was used to determine prognostic factors for risk of recurrence. The hazard rate ratio's (sHR) for the final model including the selected prognostic factors were presented. The observed 5‐year risks of recurrence for all combination of the selected prognostic variables were determined, categorized and presented in a flow chart. Logistic regression was performed to identify prognostic factors for the probability of curative‐intent treatment. For the final model including the selected factors, the odds ratios (OR) were presented, and the observed proportion of second events treated with curative intent categorized. The potential prognostic factors studied are presented in the (Table S1).

## 3 RESULTS

### 3.1 First event

The patient, tumor, and treatment characteristic of 594 patients treated with curative intent for primary OSCC (first event) are displayed in Table 1.

**Table 1:** Patient, tumor and treatment characteristics, and survival and recurrence rates of patients treated for primary (*N* = 594) and recurrent (*N* = 106) OSCC with curative intent

		First event (*N* = 594)		Second event (*N* = 106)	
		No	%	No	%
Overall		594		106	
*Patient characteristics*					
Gender	Male	359	60%	62	58%
	Female	235	40%	44	42%
Age at diagnosis	<40 years	24	4%	6	6%
	40‐60 years	237	40%	32	30%
	≥60 years	333	56%	68	64%
ASA score at primary diagnosis	I	145	24%	29	27%
	II	323	54%	59	56%
	III	96	16%	13	12%
	IV	2	1%	–	–
	Unknown	28	5%	5	5%
Malignancies in the past (primary diagnosis)	Yes	35	6%	2	2%
	No	559	94%	104	98%
Oral premalignancies in the past (primary diagnosis)	Yes	27	4%	12	11%
	No	564	95%	94	89%
	Unknown	3	1%	–	–
Karnofsky Performance Score at primary diagnosis	<60	3	1%	–	–
	60	13	2%	–	–
	70	18	3%	2	2%
	80	32	6%	7	7%
	90	79	13%	12	11%
	100	55	9%	16	15%
	Unknown	394	66%	69	65%
Smoking and alcohol at primary diagnosis	Never smoker, none‐moderate alcohol use	96	16%	25	24%
	Never smoker, problematic alcohol use	3	1%	0	–
	(ex) smoker, none‐moderate alcohol use	217	36%	38	36%
	(ex) smoker, problematic alcohol use	206	35%	31	29%
	Unknown	72	12%	12	11%
*Tumor characteristics*					
Tumor stage	1	222	37%	36	34%
	2	214	36%	10	9%
	3	45	8%	5	5%
	4 (a+b)	113	19%	5	5%
	Unknown	–	–	50	47%
Nodal stage	0	368	62%	55	52%
	1	68	12%	17	16%
	2	127	21%	10	9%
	Unknown	31	5%	24	23%
Stage	1	177	30%	33	31%
	2	129	22%	8	8%
	3	68	11%	9	8%
	4	189	32%	6	6%
	Unknown	31	5%	50	47%
Location	Tongue	216	36%	20	19%
	Buccal mucosa	48	8%	9	8%
	Floor of the mouth	208	35%	16	15%
	Retromolar trigone	52	9%	4	4%
	Alveolar process	66	11%	14	13%
	Other	4	1%	43	41%
*Treatment characteristics*					
Therapy	Surgery only	260	44%	53	50%
	Radiotherapy only	11	2%	8	8%
	Surgery and radiotherapy	276	46%	28	26%
	Surgery and chemoradiation	27	5%	8	8%
	Chemoradiation	20	3%	6	5%
	Chemotherapy only	–		2	2%
	Surgery and chemotherapy	–		1	1%
Surgery	Yes	563	95%	89	84%
	No	31	5%	17	16%
Optimal treatment as advised by the multi disciplinary team meeting	Yes	509	86%	82	77%
	No	78	13%	22	21%
	Unknown	7	1%	2	2%
*Follow‐up characteristics*					
Follow‐up time	Median (range) years	7.8 (0.1‐14.5)		6.0 (0.1‐13.0)	
5‐year overall survival	% (95% CI)	65% (61%;69%)		64% (52%;74%)	
5‐year CIF recurrence	% (95% CI) recurrence	30% (26%;33%)		36% (26%;46%)	
5‐year CIF competing event	% (95% CI) intercurrent death	17% (14%;20%)		21% (13%;31%)	

#### 3.1.1 Risk of a second event

**Figure 1:**
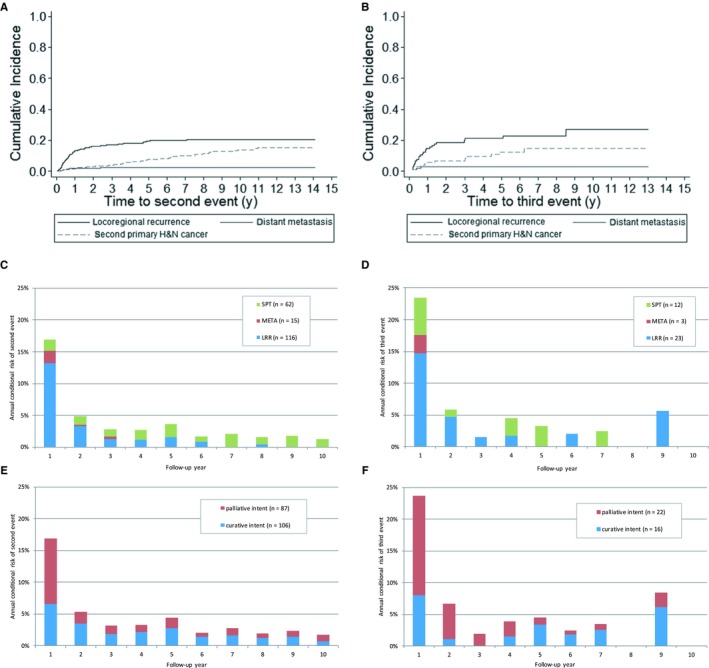
Cumulative risk of second event (A,B), annual conditional risk of second event by event type (C,D) and treatment intent (E,F). (A) Cumulative risk of a second event, by event type. (B) Cumulative risk of a third event, by event type. (C) Annual conditional risk of a second event, by event type. (D) Annual conditional risk of a third event, by event type. (E) Annual conditional risk of a second event, by treatment intent. (F) Annual conditional risk of a third event, by treatment intent

The 1‐, 5‐, and 10‐year cumulative risks of a second event (ie recurrence, new primary tumor or DM) were 17% (CI:14%;20%), 30% (CI:26%;33%) and 37% (CI:32%;41%). The majority of LRR occurred within the first year after treatment, and all the DMs within 3 years. The incidence rate of SPTs was stable over the entire follow‐up period (Figure 1A).

Annual *conditional* risk of a second event was highest in the first year of follow‐up. that is, 17% and decreasing in the following years (Figure 1C). Annual conditional risks of a second event were higher for the nonsurgically treated group compared with the surgically treated group (Figure S1).

**Figure 2:**
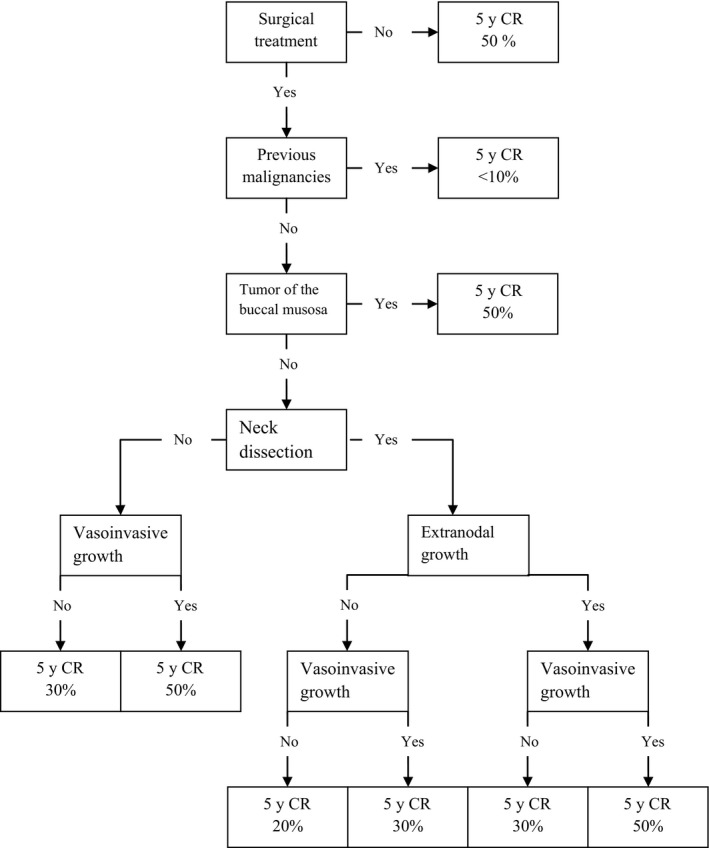
Flow chart for the observed 5‐year cumulative risk of a second event after curative‐intent treatment for OSCC

Surgical primary treatment was a statistically significant prognostic factor for risk of a second event (*P*‐value Gray test: <0.01). The 5‐year cumulative risk was 28% (CI:25%;32%) after surgical treatment and 50% (CI:31%;67%) after nonsurgical treatment. In patients treated surgically, vasoinvasive growth, cervical lymph node dissection, buccal mucosa, and extranodal growth were important independent prognostic factors for risk of second event (Table 2). Based on these factors, a flowchart was built with corresponding 5‐year cumulative risks of a second event (Figure 2). Nine risk groups, with an observed 5‐year risk of second event varying between <10% for patients who received surgical treatment and had a previous malignancy and 50%, for patients without surgical treatment were identified. The group size of the nonsurgically treated group did not permit further risk stratification, but were considered as separate risk group.

**Table 2:** Independent prognostic factors for the risk of recurrence after curative‐intent surgical treatment for primary OSCC: results from the forward selection procedure

Prognostic factor	sHR (95% CI)
Vasoinvasive growth (yes vs no)	1.6 (1.1; 2.2)
Cervical node dissection (yes vs no)	0.6 (0.4; 0.8)
Buccal mucosa (vs all other locations)	2.1 (1.3; 3.3)
Extranodal growth (yes vs no)	1.6 (1.0; 2.5)

#### 3.1.2 Treatment intent

The proportion of the 193 second events that could be treated with curative intent increased with follow‐up time from 32% (CI:20%;45%) for early recurrences (0‐6 months after treatment) to 71% (CI:57%;69%) for patients who had a second event 24‐60 months after treatment. The annual conditional risk of a second event that could not be treated curatively was highest in the first year after treatment (Figure 1E). The proportion of patients with a LRR that could be treated curatively decreased, while the proportion of patients with a SPT that could be treated curatively was stable over time.

Patients having their recurrence detected after primary surgical treatment had a higher chance of curative treatment of the second event when compared with recurrences detected after non‐surgical treatment: 58% (CI:50%;65%) vs 25% (CI:10%;50%). In patients treated surgically, postoperative radiotherapy for the first event, tumor size, nodal status, ASA‐score, and invasion depth were important independent prognostic factors for curative‐intent treatment of a second event (Table 3). Based on the number of risk factors, the chance of curative intent treatment varied between 0% (four risk factors) and 96% (no risk factors) (Table 4).

**Table 3:** Independent prognostic factors for the treatment intent of second events after curative‐intent surgical treatment for primary OSCC: results from the forward selection procedure

Prognostic factor	OR (95% CI)
Radiotherapy (yes vs no)	0.1 (0.0; 0.4)
Nodal stage 2 (vs stage 0 or 1)	0.2 (0.1; 0.5)
Tumor stage 4 (vs stage 1‐3)	0.1 (0.0; 0.5)
ASA III or IV (vs ASA I or II)	0.1 (0.0; 0.4)
Invasion dept ≥4 mm (vs <4 mm)	0.3 (0.1;1.3)

**Table 4:** The observed proportion of patients with their second event treated with curative intent related to the number of risk factors, relating to the first primary OSCC (radiotherapy, pN_2_, pT_4_, ASA3/4, invasion depth >4 mm)

Number of risk factors	Number of patients	Observed % curative intent	(95% CI)
0	27	96%	81%‐100%
1	30	97%	83%‐100%
2	39	62%	45%‐77%
3	38	26%	13%‐43%
4	12	0%	0%‐26%^a^
Missing information on one or more risk factors	31	42%	25%‐61%

a One‐sided, 97.5% confidence interval.

### 3.2 Second event

The characteristics of the 106 patients curatively treated for a second event are summarized in Table 1. The 5‐year OS rate after completion of the treatment of the second event was 49% (CI:38%;59%).

#### 3.2.1 Risk of a third event

The 1‐, 5‐, and 10‐year cumulative risks of a third event were 23% (CI:16;32%), 37% (CI:27%;47%), and 45% (CI:32%;57%). Almost all local and/or regional recurrences occurred within 2 years after treatment (Figure 1B). The risk of a new primary tumor as third event was constant over time.

The annual *conditional* risk of a third event was 24% in the first year after treatment, 7% in the second year and decreased to 4% in the fifth year after treatment (Figure 1D).

The risk for a third event did not significantly differ between patients treated with (*n* = 88) and without (*n* = 17) surgery for their second event (*P*‐value Gray test: 0.42). The number of third events was too small (*n* = 38) for a reliable search for independent prognostic factors. However, univariable analyses for prognostic factors for the risk of a third event showed similar trends when compared with univariable analyses for the risk of a second event (Table S1).

#### 3.2.2 Treatment intent

Of the 38 third events, 16 (42%) could be treated with curative intent (Figure 1F). SPTs were more often treated with curative intent (58%) than LRRs (39%). None of the three DMs were treated with curative intent. Patient numbers were too small to draw conclusions about the trends in time or to reliably compare prognostic factors for treatment intent for second vs third events. Patient‐ and tumor‐related factors for second and third events and their relationship with treatment intent are presented in the appendix (Table S2).

## 4 DISCUSSION

This study is the first comprehensive analysis of patterns of occurrence of new disease that focuses specifically on OSCC. First and second events which include both recurrences and SPTs in a large cohort with a long follow‐up time are described. The cumulative risk of recurrence for both surgically and nonsurgically treated patients was highest in the first year after treatment. Almost all LRRs occurred in the first 2 years after treatment. The incidence of SPTs was stable over the years. The time patterns of presentation of third events did not differ from that of second events. Our results are clinically highly relevant, because these patterns are not adequately reflected in the current guidelines for routine follow‐up after OSCC treatment which advocate 5 year or lifelong follow‐up after the treatment for OSCC.^15^, ^22^ Determining the optimal follow‐up schedule is very important from a patient's perspective, because unnecessary follow‐up will create unnecessary anxiety and false expectations.

Our results are consistent with the results from other authors who reported that 86%‐94% of new disease after curatively treated OSCC occurred within 2 years.^11^, ^12^, ^23^, ^24^ Consistent with Vaamonde et al we confirm that the risk for a SPT is stable over time.^25^

Arguments for lifelong follow‐up are based on the assumption that early, asymptomatic, detection of new primary tumors leads to improved survival. The literature, which comprises all HNCs rather than the oral cavity alone, remains equivocal on this subject.^26^ Site‐specific studies on laryngeal carcinomas and early‐stage OSCC did not show survival benefits.^27^ The proportion of patients treated with curative intent in our study did not differ between SPTs detected within 5 years (ie during the follow‐up period) and those detected after 5 years, suggesting that it is safe to shorten the follow‐up period. When shortening the follow‐up program, it is important to educate patients about the symptoms of new disease and provide them easy access to the clinic in case of symptoms.^28^

If follow‐up is proven to be beneficial to patients, customization of follow‐up schedules based on risk of recurrence can be beneficial. Using 6 independent prognostic factors, namely surgical treatment, previous malignancy, presence of vasoinvasive growth, neck dissection, localization of the tumor and the presence of extranodal growth, we were able to identify patient groups with 5‐year risks of a second event varying between <10% and 50%. The prognostic value of these factors has been confirmed by several other authors.^29^, ^30^ In our patient group the risk of recurrence between the different locations differed significantly (*P* < 0.01). The literature concerning the effect of location on the risk of recurrence is not unequivocal, with some authors reporting a significant effect on the risk of recurrence^31^, ^32^ and others not.^33^, ^34^ This reflects the complex multifactorial nature of oral cancer, which goes beyond purely anatomical factors.

Of interest is also that patients with a previous malignancy have a statistically significant lower risk of second events. This is likely to be caused by the fact that they have a higher risk of intercurrent death from other causes (data not shown). Another important prognostic factor for a second event was if the patient underwent an elective neck dissection for the treatment of their first primary tumor. This can partially be explained by the fact that patients with a clinically negative neck, a small tumor and an invasion depth <4 mm did not undergo an elective neck dissection. Montero et al built a nomogram predicting the probability of LRR‐free survival comprising nodal status, the subsites, bone invasion and primary tumor size.^35^ These parameters largely overlap ours. If routine follow‐up is considered effective, the nomograms for the risk of a second event might aid the development of a personalized follow‐up program, but should undergo further detailed evaluation and validation.

Another way to personalize follow‐up is by considering the chance of curative intent treatment of a second event. This strategy has been advocated by Kanatas et al, who suggested that patients with early disease who were treated with a single modality, might benefit from earlier discharge.^36^ Patients who develop DM will have no chance to be cured. The likelihood of curative intent treatment of the second event could be predicted using 5 factors, that is previous radiotherapy, nodal status of the first primary tumor, tumor size, invasion depth, and ASA‐score resulting in observed probabilities ranging between 0% and 96%. The factors associated with a treatment with curative intent are all related to the possibilities a patient has left to receive therapy. Many patients will have undergone treatment for the neck consisting of neck dissection and/or (chemo)radiotherapy.^4^ Several authors confirmed that patients with previous neck dissections had a markedly smaller chance of successful salvage surgery.^24^, ^37^, ^38^ Likewise, in most patients who had previous radiotherapy another course of radiotherapy will not be possible.^39^

Other authors mentioned performance status, ASA‐score and previous quality of life as important factors for a successful salvage.^9^ Our results confirm that time to recurrence yields important prognostic information for the success of salvage.^24^, ^40^, ^41^ This is the first attempt to determine subgroups of patients for whom curative treatment of second events may be available. Patients who are unlikely to be treated curatively for their next event might benefit from a follow‐up program that focuses more on quality of life than on the early detection of new disease.

A limitation of this study is that changes in patient‐related factors such as smoking, alcohol use and ASA‐score could not be taken into account as these data were only available at the time of the first diagnosis.^42^ As the Karnofsky score was only available in 44% of the patients, we could not include this parameter in our prediction models.

Strengths of this study are the large, site‐specific patient cohort that was followed by a strict protocol with very high compliance rates and the description both first and second events. By the use of competing‐risk analysis, a more accurate estimation of absolute risks is given than the Kaplan‐Meier method which usually overestimates the cumulative risk of events when competing risks, like mortality, occur.

Our study shows that a 2‐year follow‐up period is sufficient for the detection of LRRs. Longer follow‐up may be indicated on an individual basis for treatment‐related morbidities and dental rehabilitation.^43^, ^44^ We therefore advocate a personalized follow‐up schedule with a “core follow‐up” for 2 years after which frequency, type of clinician and duration are tailored to the patient's needs. In order to timely diagnose new disease after discharge, patients should be educated to recognize symptoms of new disease.^36^

Our findings support a follow‐up time of 2 years after curative‐intent treatment for OSCC. Longer follow‐up may be needed for some individual patients due to treatment‐related morbidities and psychological needs. Based on the patterns of occurrence of third events, a separate follow‐up protocol after curative treatment of a second event is not needed. The 2 prediction models developed in this study might, after validation, be a good starting point when personalizing OSCC follow‐up. In order to further optimize the guidelines for follow‐up and determine the optimal duration of follow‐up future research should focus on elucidating the benefits and risks of risk‐stratified follow‐up and its influence on survival or quality of life.

## CONFLICT OF INTEREST

The authors declare no conflict of interest.

## References

[1] Karim‐Kos HE , de Vries E , Soerjomataram I , Lemmens V , Siesling S , Coebergh JW . Recent trends of cancer in Europe: a combined approach of incidence, survival and mortality for 17 cancer sites since the 1990s. Eur J Cancer. 2008;44:1345‐1389. 18280139 10.1016/j.ejca.2007.12.015

[2] Siegel RL , Miller KD , Jemal A . Cancer statistics, 2016. CA Cancer J Clin. 2016;66:7‐30. 26742998 10.3322/caac.21332

[3] van Dijk BA , Brands MT , Geurts SM , Merkx MA , Roodenburg JL . Trends in oral cavity cancer incidence, mortality, survival and treatment in the Netherlands. Int J Cancer. 2016;139:574‐583. 27038013 10.1002/ijc.30107

[4] Rogers SN , Brown JS , Woolgar JA , et al. Survival following primary surgery for oral cancer. Oral Oncol. 2009;45:201‐211. 18674959 10.1016/j.oraloncology.2008.05.008

[5] Mucke T , Wagenpfeil S , Kesting MR , Holzle F , Wolff KD . Recurrence interval affects survival after local relapse of oral cancer. Oral Oncol. 2009;45:687‐691. 19095488 10.1016/j.oraloncology.2008.10.011

[6] Brands MT , Brennan PA , Verbeek ALM , Merkx MAW , Geurts SME . Follow‐up after curative treatment for oral squamous cell carcinoma. A critical appraisal of the guidelines and a review of the literature. Eur J Surg Oncol. 2018;44:559‐565. 29433990 10.1016/j.ejso.2018.01.004

[7] Boysen M , Lovdal O , Tausjo J , Winther F . The value of follow‐up in patients treated for squamous cell carcinoma of the head and neck. Eur J Cancer. 1992;28:426‐430. 1591057 10.1016/s0959-8049(05)80068-1

[8] Kothari P , Trinidade A , Hewitt RJD , Singh A , O'Flynn P . The follow‐up of patients with head and neck cancer: an analysis of 1,039 patients. Eur Arch Otorhinolaryngol. 2011;268:1191‐1200. 21193920 10.1007/s00405-010-1461-2

[9] Ho AS , Kraus DH , Ganly I , Lee NY , Shah JP , Morris LG . Decision making in the management of recurrent head and neck cancer. Head Neck. 2014;36:144‐151. 23471843 10.1002/hed.23227

[10] Liu CH , Chen HJ , Wang PC , Chen HS , Chang YL . Patterns of recurrence and second primary tumors in oral squamous cell carcinoma treated with surgery alone. Kaohsiung J Med Sci. 2013;29:554‐559. 24099110 10.1016/j.kjms.2013.03.001 PMC11916622

[11] Merkx MA , van Gulick JJ , Marres HA , et al. Effectiveness of routine follow‐up of patients treated for T1‐2N0 oral squamous cell carcinomas of the floor of mouth and tongue. Head Neck. 2006;28:1‐7. 16155911 10.1002/hed.20296

[12] Sasaki M , Aoki T , Karakida K , et al. Postoperative follow‐up strategy in patients with oral squamous cell carcinoma. J Oral Maxillofac Surg. 2011;69:e105‐e111. 21419541 10.1016/j.joms.2010.11.039

[13] Taslim SJ , Leemans CR , van der Waal I , Karagozoglu KH . Follow‐up of oral cancer patients: three uneventful years may be enough. Oral Surg Oral Med Oral Pathol Oral Radiol. 2016;122:434‐439. 27496578 10.1016/j.oooo.2016.05.009

[14] Crawford B , Greenberg DD . Patient Surveillance after Cancer Treatment. New York: Springer; 2013.

[15] Nederlandse Werkgroep Hoofd-halstumoren . Richtlijn Mondholte‐ en Orofarynxcarcinoom Alphen aan den Rijn: Van Zuiden communications B.V. 2004.

[16] Flynn CJ , Khaouam N , Gardner S , et al. The value of periodic follow‐up in the detection of recurrences after radical treatment in locally advanced head and neck cancer. Clin Oncol (R Coll Radiol). 2010;22:868‐873. 20650620 10.1016/j.clon.2010.05.016

[17] Cooney TR , Poulsen MG . Is routine follow‐up useful after combined‐modality therapy for advanced head and neck cancer? Arch Otolaryngol Head Neck Surg. 1999;125:379‐382. 10208674 10.1001/archotol.125.4.379

[18] Warren S , Gates L . Multiple malignant tumors, a survey of the literature and statistical study. Am J Cancer. 1932;16:1358‐1414.

[19] Curtis R , Ries L . New Malignancies among Cancer Survivors: SEER Cancer Registries 1973–2000. Bethesda: National Cancer Institute; 2006:9‐14.

[20] Coviello V , Boggess M . Cumulative incidence estimation in the presence of competing risks. Stata J. 2004;4:103‐112.

[21] Skuladottir H , Olsen JH . Conditional survival of patients with the four major histologic subgroups of lung cancer in Denmark. J Clin Oncol. 2003;21:3035‐3040. 12915592 10.1200/JCO.2003.04.521

[22] NCCN . NCCN Clinical Practice Guidelines in Oncology. Version 2.2016 — October 11, 2016; 2016.

[23] Kissun D , Magennis P , Lowe D , Brown JS , Vaughan ED , Rogers SN . Timing and presentation of recurrent oral and oropharyngeal squamous cell carcinoma and awareness in the outpatient clinic. Br J Oral Maxillofac Surg. 2006;44:371‐376. 16624459 10.1016/j.bjoms.2005.08.010

[24] Kowalski LP . Results of salvage treatment of the neck in patients with oral cancer. Arch Otolaryngol Head Neck Surg. 2002;128:58‐62. 11784256 10.1001/archotol.128.1.58

[25] Vaamonde P , Martin C , del Rio M , LaBella T . Second primary malignancies in patients with cancer of the head and neck. Otolaryngol Head Neck Surg. 2003;129:65‐70. 12869919 10.1016/S0194-59980300476-5

[26] Liu G , Dierks EJ , Bell RB , Bui TG , Potter BE . Post‐therapeutic surveillance schedule for oral cancer: is there agreement? Oral Maxillofac Surg. 2012;16:327‐340. 22941063 10.1007/s10006-012-0356-3

[27] Ritoe SC , Krabbe PF , Kaanders JH , van den Hoogen FJ , Verbeek AL , Marres HA . Value of routine follow‐up for patients cured of laryngeal carcinoma. Cancer. 2004;101:1382‐1389. 15368326 10.1002/cncr.20536

[28] De Zoysa N , Lee A , Joshi A , et al. Developing a follow‐up surveillance protocol in head and neck oncological surgery: enhanced ‘traffic light’ surveillance ‐ a prospective feasibility study. Clin Otolaryngol. 2017;42:446‐450. 26696519 10.1111/coa.12613

[29] Jung YH , Song CM , Park JH , et al. Efficacy of current regular follow‐up policy after treatment for head and neck cancer: need for individualized and obligatory follow‐up strategy. Head Neck. 2014;36:715‐721. 23616261 10.1002/hed.23364

[30] Agrawal A , Hammond TH , Young GS , Avon AL , Ozer E , Schuller DE . Factors affecting long‐term survival in patients with recurrent head and neck cancer may help define the role of post‐treatment surveillance. Laryngoscope. 2009;119:2135‐2140. 19507214 10.1002/lary.20527

[31] Fan KH , Wang HM , Kang CJ , et al. Treatment results of postoperative radiotherapy on squamous cell carcinoma of the oral cavity: coexistence of multiple minor risk factors results in higher recurrence rates. Int J Radiat Oncol Biol Phys. 2010;77:1024‐1029. 20610038 10.1016/j.ijrobp.2009.06.064

[32] Brandwein‐Gensler M , Teixeira MS , Lewis CM , et al. Oral squamous cell carcinoma: histologic risk assessment, but not margin status, is strongly predictive of local disease‐free and overall survival. Am J Surg Pathol. 2005;29:167‐178. 15644773 10.1097/01.pas.0000149687.90710.21

[33] Huang TY , Hsu LP , Wen YH , et al. Predictors of locoregional recurrence in early stage oral cavity cancer with free surgical margins. Oral Oncol. 2010;46:49‐55. 20005769 10.1016/j.oraloncology.2009.10.011

[34] Ghantous Y , Bahouth Z , Abu El‐Naaj I . Clinical and genetic signatures of local recurrence in oral squamous cell carcinoma. Arch Oral Biol. 2018;95:141‐148. 30118965 10.1016/j.archoralbio.2018.07.018

[35] Montero PH , Yu C , Palmer FL , et al. Nomograms for preoperative prediction of prognosis in patients with oral cavity squamous cell carcinoma. Cancer. 2014;120:214‐221. 24399417 10.1002/cncr.28407

[36] Kanatas A , Bala N , Lowe D , Rogers SN . Outpatient follow‐up appointments for patients having curative treatment for cancer of the head and neck: are the current arrangements in need of change? Br J Oral Maxillofac Surg. 2014;52:681‐687. 25037165 10.1016/j.bjoms.2014.06.017

[37] Wong LY , Wei WI , Lam LK , Yuen AP . Salvage of recurrent head and neck squamous cell carcinoma after primary curative surgery. Head Neck. 2003;25:953‐959. 14603456 10.1002/hed.10310

[38] Lim JY , Lim YC , Kim SH , Byeon HK , Choi EC . Factors predictive of successful outcome following salvage treatment of isolated neck recurrences. Otolaryngol Head Neck Surg. 2010;142:832‐837. 20493354 10.1016/j.otohns.2010.01.024

[39] Argiris A , Li Y , Forastiere A . Prognostic factors and long‐term survivorship in patients with recurrent or metastatic carcinoma of the head and neck. Cancer. 2004;101:2222‐2229. 15452834 10.1002/cncr.20640

[40] Goodwin WJ Jr . Salvage surgery for patients with recurrent squamous cell carcinoma of the upper aerodigestive tract: when do the ends justify the means? Laryngoscope. 2000;110:1‐18. 10.1097/00005537-200003001-00001 10714711

[41] Kernohan MD , Clark JR , Gao K , Ebrahimi A , Milross CG . Predicting the prognosis of oral squamous cell carcinoma after first recurrence. Arch Otolaryngol Head Neck Surg. 2010;136:1235‐1239. 21173373 10.1001/archoto.2010.214

[42] Bosetti C , Gallus S , Peto R , et al. Tobacco smoking, smoking cessation, and cumulative risk of upper aerodigestive tract cancers. Am J Epidemiol. 2008;167:468‐473. 18056925 10.1093/aje/kwm318

[43] Pagh A , Grau C , Overgaard J . A longitudinal study of follow‐up activities after curative treatment for head and neck cancer. Acta Oncol. 2015;54:813‐819. 25907822 10.3109/0284186X.2015.1028591

[44] Wetzels JW , Koole R , Meijer GJ , de Haan AF , Merkx MA , Speksnijder CM . Functional benefits of implants placed during ablative surgery: a 5‐year prospective study on the prosthodontic rehabilitation of 56 edentulous oral cancer patients. Head Neck. 2016;38(Suppl 1):E2103‐E2111. 26873437 10.1002/hed.24389

