# Supplementary figures and images for: Time patterns of recurrence and second primary tumors in a large cohort of patients treated for oral cavity cancer
Source: Cancer Med. 2019 Aug 10;8(12):5810–9. doi: 10.1002/cam4.2124 (PMC6745868; doi:10.1002/cam4.2124)

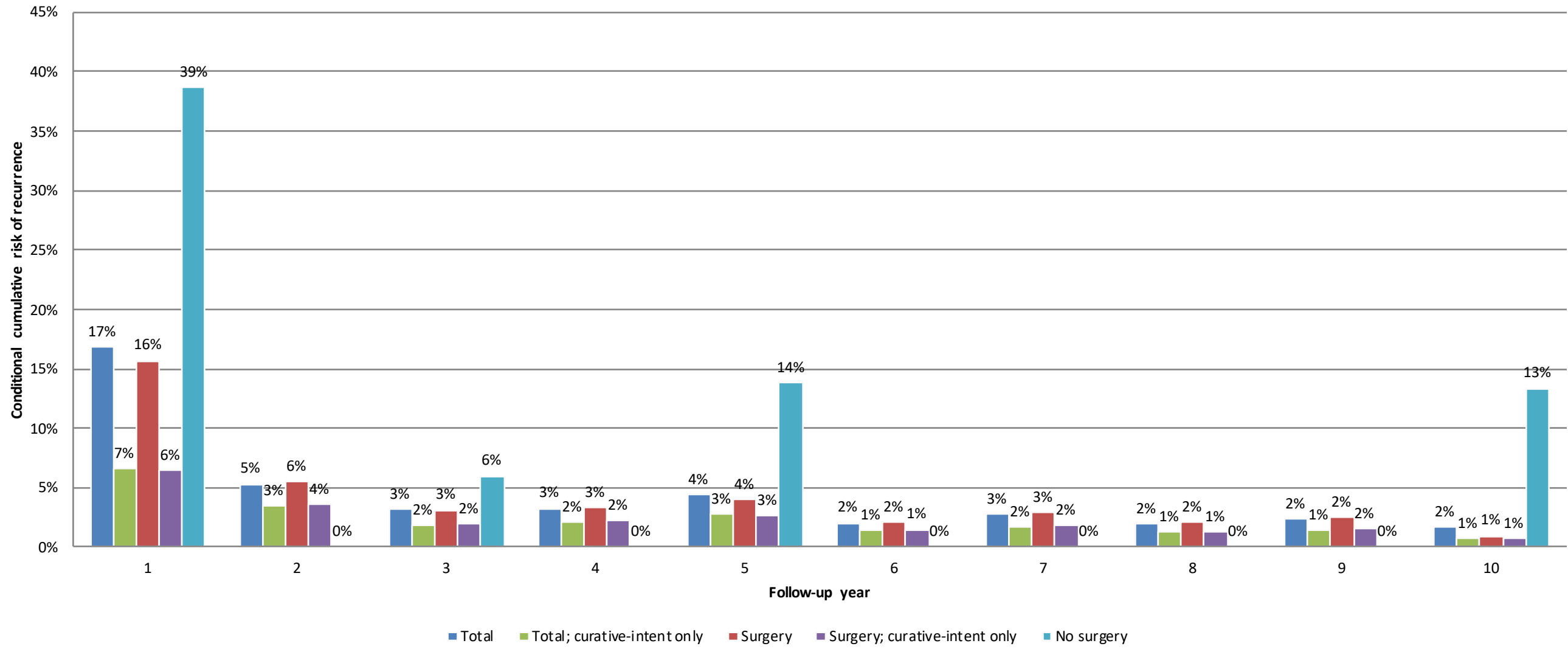

Supplement: Supplementary file 1 [file CAM4-8-5810-s001.pdf]
