# Supplementary material for: Time patterns of recurrence and second primary tumors in a large cohort of patients treated for oral cavity cancer
Source: Cancer Med. 2019 Aug 10;8(12):5810–9. doi: 10.1002/cam4.2124 (PMC6745868; doi:10.1002/cam4.2124)
Supplement: Supplementary file 2 [file CAM4-8-5810-s002.docx]

##### **Supplementary data**

##### **Table S1.** Prognostic factors for the occurrence of recurrent disease after curative-intent surgical treatment of an OSCC: univariable competing risk regression analyses after event 1 (n=563) and event 2 (n=105), respectively.

|  | | **First event (N=563)** | | | | | **Second event (N=105)** | | | | |
| --- | --- | --- | --- | --- | --- | --- | --- | --- | --- | --- | --- |
|  |  |  |  |  |  |  |  |  |  |  |  |
|  |  | **No** | **%** | **sHRR (95% CI)** | **Gray’s**  **p-value** | **5-yr risk of**  **recurrence** | **No** | **%** | **sHRR (95% CI)** | **Gray’s**  **p-value** | **5-yr risk of**  **recurrence** |
| **Patient characteristics** | |  |  |  |  |  |  |  |  |  |  |
| Gender | Male  Female | 336  227 | 60%  40% | Ref.  1.1 (0.8; 1.5) | 0.51 | 28%  29% | 62  43 | 59%  41% | Ref.  1.6 (0.9; 3.1) | 0.32 | 29%  48% |
| Age | <40 years  40-59 years  ≥60 years | 24  225  314 | 4%  40%  56% | 1.7 (0.8; 3.5)  Ref.  1.4 (1.1;2.0) | <0.01 | 40-59: 23%  <40\|≥60: 32% | 7  45  53 | 6%  43%  51% | 0.7 (0.2; 3.2)  Ref.  1.0 (0.5; 1.8) | 0.83 | 40-59: 36%  <40\|≥60: 36% |
| ASA score | I  II  III or IV  Unknown | 137  310  92  24 | 24%  55%  16%  4% | Ref.  1.2 (0.8; 1.8)  1.2 (0.7; 2.0) | 0.25 | 25%  28%  30% | 29  58  13  5 | 28%  55%  12%  5% | Ref.  1.5 (0.7; 3.3)  1.6 (0.5; 5.4) | 0.25 | 30%  38%  38% |
| Malignancies in the past | No  Yes | 530  33 | 94%  6% | Ref.  **0.2 (0.1;0.8)*** | 0.03 | 30%  9% | 103  2 | 98%  2% | Ref.  1.4 (0.2; 7.9) | 0.59 | 50%  36% |
| Smoking and alcohol | Never smoker, none-moderate alcohol use  (Ex) smoker, none-moderate alcohol use  Problematic alcohol use  Unknown | 95  204  195  69 | 17%  36%  35%  12% | Ref.  0.8 (0.5; 1.2)  **0.6 (0.4; 1.0)*** | 0.16 | 34%  28%  25% | 25  37  31  12 | 24%  35%  30%  11% | Ref.  1.1 (0.5; 2.7)  0.6 (0.2; 1.6) | 0.54 | 43%  40%  25% |
| **Tumor characteristics** | |  |  |  |  |  |  |  |  |  |  |
| Location | Tongue  Buccal mucosa (BM)  Floor of the mouth  Retromolar trigone  Alveolar process  Other | 212  45  195  43  64  4 | 38%  8%  35%  8%  11%  1% | Ref.  **2.3 (1.4;3.7)***  1.0 (0.7; 1.5)  0.9 (0.5; 1.7)  1.7 (1.1; 2.6)  0.9 (0.1; 7.4) | <0.01 | BM: 48%  Other: 27% | 20  8  16  4  14  43 | 19%  8%  15%  4%  13%  41% | Ref.  **3.0 (1.1; 8.4)***  0.8 (0.2; 2.9)  1.1 (0.4; 2.7)  1.5 (0.5; 4.4)  N.A. | 0.15 | BM: 88%  Other: 32% |
| Pathological T-stage | 1  2  3  4 (a+b)  Unknown | 221  210  37  95  - | 39%  37%  7%  17% | Ref.  1.0 (0.7; 1.5)  0.9 (0.5; 1.9)  1.4 (0.9; 2.1) | 0.21 | 27%  27%  22%  36% | 36  9  5  5  50 | 34%  9%  5%  5%  48% | Ref.  **3.6 (1.4; 9.4)***  1.3 (0.3; 6.1)  2.1 (0.5; 9.7) | 0.05 | 32%  80%  40%  50% |
| Pathological N-stage | 0  1  2  Unknown | 368  68  127 | 65%  12%  23% | Ref.  1.0 (0.6; 1.7)  **1.6 (1.1; 2.3)*** | <0.01 | 26%  27%  37% | 54  17  10  24 | 51%  16%  10%  23% | Ref.  0.5 (0.2; 1.3)  1.2 (0.4; 4.0) | 0.40 | 46%  27%  40% |
| Optimal treatment | Yes  No  Unknown | 504  54  5 | 90%  10%  1% | Ref.  **2.0 (1.3; 3.1)*** | <0.01 | 27%  51% | 81  22  2 | 77%  21%  2% | Ref.  **1.8 (0.9; 3.5)*** | 0.02 | 32% 73% |
| Surgery | Yes  No | N.A. |  |  |  |  | 88  17 | 84%  16% | Ref.  0.4 (0.1; 1.5) | 0.42 | 40%  19% |
| **Histological characteristics** | |  |  |  |  |  |  |  |  |  |  |
| Differentiation tumor | Well  Moderate  Poor  Unknown | 85  369  78  31 | 15%  66%  14%  6% | Ref.  0.8 (0.6; 1.2)  1.1 (0.6; 1.8) | 0.45 | 33%  26%  34% | 14  37  16  38 | 13%  35%  15%  36% | Ref.  2.1 (0.7; 6.7)  1.9 (0.5; 7.2) | 0.37 | 31%  51%  44% |
| Resection margins | Negative margins  Positive margins  Unknown | 460  87  16 | 83%  15%  3% | Ref  1.6(1.1;2.4) | 0.44 | 26%  38% | 47  14  44 | 45%  13%  42% | Ref  5.4 (2.5;11.6) | <0.001 | 26%  86% |
| Perineural growth | No  Yes  No surgery  Unknown | 338  181  N.A.  44 | 60%  32%  8% | Ref.  1.3 (0.9; 1.7) | 0.09 | 26%  32% | 38  9  41  17 | 36%  9%  39%  16% | Ref.  **3.0 (1.2; 7.6)***  0.8 (0.4; 1.8) | 0.05 | 38%  56%  27% |
| Vasoinvasive growth | No  Yes  No surgery  Unknown | 351  164  N.A.  48 | 62%  29%  9% | Ref.  **1.5 (1.1; 2.1)*** | <0.01 | 25%  35% | 36  8  41  20 | 34%  8%  39%  19% | Ref.  **3.4 (1.3; 8.9)***  0.8 (0.4; 1.8) | 0.02 | 39%  63%  27% |
| Spidery growth | No  Yes  No surgery  Unknown | 339  166  N.A.  58 | 60%  29%  11% | Ref.  1.0 (0.7; 1.4) | 0.85 | 27%  27% | 29  13  41  22 | 28%  12%  39%  21% | Ref.  **2.3 (0.9; 5.6)***  1.0 (0.4; 2.2) | 0.11 | 36%  54%  27% |
| Bone invasion | No  Yes  No surgery  Unknown | 476  85  N.A.  2 | 85%  15%  0% | Ref.  1.3 (0.9; 1.9) | 0.11 | 27%  33% | 57  4  40  4 | 54%  4%  38%  4% | Ref.  **3.2 (1.2; 8.9)***  0.7 (0.3; 1.4) | 0.09 | 41%  67%  25% |
| Extranodal growth | No  Yes  No cervical node dissection  Unknown | 379  81  100  3 | 68%  14%  18%  0% | Ref.  **1.7 (1.1; 2.5)***  **1.6 (1.1; 2.3)*** | <0.01 | 25%  37%  35% | 31  21  47  6 | 30%  50%  45%  5% | Ref.  0.7 (0.3; 1.9)  0.7 (0.4; 1.5) | 0.64 | 40%  34%  37% |
| Invasion depth in mm | <4mm  ≥4mm  Unknown | 117  388  58 | 21%  69%  10% | Ref.  0.8 (0.5; 1.1) | 0.24 | 32%  26% | 17  25  63 | 16%  24%  60% | Ref.  **2.4 (0.9; 6.7)*** | 0.08 | 25%  58% |

*p<0.10

N.A. not applicable

**Table S2:** Univariable logistic regression for treatment intent at time of recurrence of an OSCC (curative 1, palliative 0), in initially surgically treated patients with a second event and all relapsed patients with a third event.

|  | | **Treatment intent second event (N=177)** | | | | **Treatment intent third event (N=38)** | | | |
| --- | --- | --- | --- | --- | --- | --- | --- | --- | --- |
|  |  |  | **N** | **(%) curative** | **Univariable** |  | **N** | **(%) curative** | **Univariable** |
|  |  |  |  |  | **OR (95% CI)** |  |  |  | **OR (95% CI)** |
| Total |  | 177 | 102 | 58% |  | 38 | 16 | 41% |  |
| **Patient characteristics** | | | | | | | | | |
| Gender | Male Female | 102 75 | 58  44 | 57% 59% | Ref. 1.1 (0.6; 2.0) | 18 20 | 6  10 | 33% 50% | Ref. 2.0 (0.5; 7.4) |
| Age | <40years 40-60years ≥60years | 9 61 107 | 7  43  52 | 78% 70% 49% | 1.5 (0.3; 7.7) Ref. **0.4 (0.2; 0.8)*** | 2 16 20 | 1  6  9 | 50% 38% 45% | 1.7 (0.1; 31.9) Ref. 1.4 (0.4; 5.2) |
| ASA score | I II III or IV Unknown | 38 98 28 13 | 28  59  11 4 | 74% 60% 39% 31% | Ref. 0.5 (0.2; 1.2) **0.2 (0.1; 0.7)*** | 9 21 5 3 | 5  10  1  0 | 56% 48% 20% 0% | Ref. 0.7 (0.2; 3.5) 0.2 (0.0; 2.6) |
| Malignancies in the past | No Yes | 173 4 | 101  1 | 58% 25% | Ref. 0.2 (0.0; 2.3) | 37 1 | 16  0 | 43% 0% | N.A. |
| Smoking and alcohol | Never smoker, none-moderate alcohol use (Ex) smoker, none-moderate alcohol use Problematic alcohol use Unknown | 36 64 53 24 | 25  36  29 12 | 69% 56% 55% 50% | Ref. 0.6 (0.2; 1.3) 0.5 (0.2; 1.3) | 10 15 7 5 | 4  7  3 2 | 40% 47% 43% 40% | Ref. 1.3 (0.3; 6.6) 1.1 (0.2; 8.0) |
| **Tumor characteristics** | | | | | | | | | |
| LocatioN | Tongue Buccal mucosa Floor of the mouth Alveolar process  Retromolar trigone or other | 58 23 55 27 14 | 37  10  36  13  6 | 64% 43% 65% 48% 43% | Ref. **0.4 (0.2; 1.2)*** 1.1 (0.5; 2.3) 0.5 (0.2; 1.3) 0.4 (0.1; 1.4) | 6 7 4 6 15 | 3  3  0  4  6 | 50% 43% 0% 67% 40% | Ref. 0.8 (0.1; 6.7) - 2.0 (0.2; 20.6) 0.7 (0.1; 4.5) |
| Pathological T-stage | 1 2 3 4 (a+b) Unknown | 67 64 10 36 | 57  36  3  6 | 85% 56% 30% 17% | Ref. **0.2 (0.1; 0.5)*** **0.1 (0.2; 0.3)*** **0.04 (0.01; 0.1)*** | 13 6 2 2 15 | 10  0  1  0  5 | 77% 0% 50% 0% 33% | Ref. - 0.3 (0.0; 6.4) - |
| Pathological N-stage | 0 1 2 Unknown | 107 19 51 | 78 13  11 | 73% 68% 22% | Ref. 0.8 (0.3; 2.3) **0.1 (0.1; 0.2)*** | 25 5 4 4 | 12  3  1  0 | 48% 60% 25% 0% | Ref. 2.8 (0.3; 30.4) 4.5 (0.3; 80.6) |
| Optimal treatment | Yes No Unknown | 152 24 1 | 91  10  1 | 60% 42% 100% | Ref. **0.5 (0.2; 1.1)*** | 27 11 | 13  3 | 48% 27% | Ref. 2.5 (0.5; 11.4) |
| Surgery | Yes No | N.A. | N.A. | N.A. |  | 35 3 | 15  1 | 43% 33% | Ref. 0.7 (0.1; 8.1) |
| **Histological characteristics** | | | | | | | | | |
| Differentiation tumor | Well Moderate Poor Unknown | 30 110 28 9 | 19  67  12  4 | 63% 61% 43% 44% | Ref. 0.9 (0.4; 2.0) 0.4 (0.2; 1.2) | 4 20 7 7 | 2  8  2  4 | 50% 40% 29% 57% | Ref. 0.7 (0.1; 5.7) 0.4 (0.0; 5.2) |
| Resection margins | Negative margins Positive margins Unknown | 138 34 5 | 90 10  2 | 65% 29% 40% | Ref. **0.2 (0.1;0.5)*** | 17 11 10 | 9  3  4 | 53% 27% 40% | Ref. 0.3 (0.1;1.7) |
| Perineural growth | No Yes No surgery Unknown | 100 61 N.A. 16 | 74  21 7 | 74% 34% 44% | Ref. **0.2 (0.1; 0.4)*** | 15 5 11 | 7  0  5 | 47%  0% 45% | Ref. - 1.0 (0.2; 4.5) |
| Vasoinvasive growth | No Yes No surgery Unknown | 95 65 N.A. 17 | 63 31 8 | 66% 48% 47% | Ref. **0.5 (0.2; 0.9)*** | 15 5 11 | 6  2  5 | 40% 40% 45% | Ref. 1.0 (0.1; 7.9) 1.3 (0.3; 6.0) |
| Spidery growth | No Yes No surgery Unknown | 10547 N.A.25 | 73  20  9 | 70% 42% 36% | Ref. **0.3 (0.2; 0.7)*** | 10 8 11 | 5  2  5 | 50% 25% 45% | Ref. 0.3 (0.0; 2.5) 0.8 (0.1; 4.6) |
| Bone invasion | No Yes No surgery Unknown | 144 32 N.A.1 | 96  6  0 | 67% 19% 0% | Ref. **0.1 (0.0; 0.3)*** | 24 2 10 | 10  0  4 | 42%  0% 40% | Ref. - 0.9 (0.2; 4.2) |
| Extranodal growth | No Yes No cervical node dissection Unknown | 105 32 40 | 64  11  27 | 61% 35% 68% | Ref. **0.4 (0.2; 0.8)*** 1.3 (0.6; 2.9) | 15 6 15 | 7  2  7 | 47% 33% 47% | Ref. 0.6 (0.1; 4.1) 1.0 (0.2; 4.2) |
| Invasion depth in mm | <4mm ≥4mm Unknown | 43 10925 | 35 56  11 | 81% 51% 44% | Ref. **0.2 (0.1; 0.6)*** | 6 14 | 4  4 | 67% 29% | Ref. 0.2 (0.0; 1.6) |

*p<0.10
